# Head and neck related quality of life following glossectomy among tongue cancer patients: a systematic review and meta-analysis

**DOI:** 10.1007/s10006-025-01471-y

**Published:** 2025-09-30

**Authors:** Samuel Tundealao, Praise Okunlola, Orges Alabaku, Olumide Noah, Oluwaferanmi Alufa, Tolulope Titiloye

**Affiliations:** 1 Department of Biostatistics and Epidemiology, Rutgers School of Public Health, 683 Hoes Lane, Piscataway, NJ 08854 USA; 2 Cancer Health Equity Center of Excellence, Rutgers Cancer Institute, New Brunswick, NJ USA; 3 Faculty of Dentistry, College of Medicine, University of Ibadan, Ibadan, Oyo State Nigeria; 4 Rutgers Center for Pharmacoepidemiology and Treatment Science, New Brunswick, NJ USA; 5 College of Medicine, University of Lagos, Yaba, Lagos State Nigeria; 6 Mental Health Association of Morris and Sussex, Parsippany, NJ USA

**Keywords:** Tongue cancer; glossectomy, Quality of life, Flap, Reconstruction

## Abstract

**Objective:**

This study synthesized previous studies to evaluate the post-operative head and neck-related quality of life (QoL) and pre-post operative change in the QoL of tongue cancer patients who underwent glossectomy.

**Methods:**

Following the PRISMA guidelines, databases were searched on April 1, 2025, for relevant articles without date limits. Mean estimates and standardized mean differences were calculated for post-glossectomy and pre-post glossectomy changes in QoL, respectively. A random-effects model employing the restricted maximum likelihood was conducted. Effect heterogeneity was assessed using Q and I^2^.

**Results:**

A total of 56 studies (2,832 participants; mean [SD] age, 55.1 [7.5] years; 68.2% male) were included in the study. The meta-analysis estimated a pooled composite QoL score of 71.9% (95% CI: 68.3–75.4) following glossectomy. Subgroup analyses revealed that patients who underwent partial/hemi-glossectomy (vs. subtotal/near-total/total glossectomy) and primary closure (vs. flap reconstruction) have higher QoL following tongue resection. Meta-analysis of 18 studies reporting both pre- and post-glossectomy QoL found an overall stabilization in QoL (SMD: -0.22; 95% CI: -0.63 to 0.19).

**Conclusion:**

This study provides potential evidence that patients with tongue cancer generally maintain their QoL following glossectomy, particularly in key functional domains such as swallowing, speech, and taste. However, high heterogeneity necessitates cautious interpretation.

**Supplementary Information:**

The online version contains supplementary material available at 10.1007/s10006-025-01471-y.

## Introduction

Tongue cancer is a significant health burden globally, accounting for approximately 25–40% of all oral cancers [1]. Squamous cell carcinoma is the predominant histological type, strongly associated with risk factors such as tobacco use, heavy alcohol consumption, and human papillomavirus (HPV) infection [2]. Tongue cancer occurs more commonly among males, particularly from the sixth decade of life, though its incidence is increasing among younger populations in the United States and Europe [3]. Despite advances in diagnosis and treatment, the 5-year survival rate is about 70%, depending on the presence of nodal metastasis and the quality of healthcare received [4, 5].

The major treatment for tongue cancer is surgical resection. It could be a partial, hemi, subtotal, near-total, or total glossectomy depending on tumor size, location, and cancer staging [6]. Adjuvant therapies, including concomitant chemoradiotherapy, are administered postoperatively in cases with high-risk features such as extracapsular spread or advanced stage [7]. These multimodal approaches aim to optimize cancer control but could cause significant functional and psychosocial morbidity [8].

The tongue plays a major role in speech, mastication, taste perception, and swallowing [9]. Surgical resection significantly affects these functions, causing a decrease in a patient’s quality of life (QoL). To prevent these impairments, reconstructive surgery, primarily through free flap or pedicled flap techniques, is commonly employed [10]. Despite reconstruction, patients often experience varying degrees of functional limitations depending on factors like flap volume, comorbidities, and the skill of the surgical team [11].

Many studies have explored the post-glossectomy QoL with mixed findings. While some report significant impairments in swallowing, speech, and taste, commonly assessed with validated tools [12, 13], other studies suggest that even with appropriate rehabilitation and surgical techniques, patients may not have satisfactory QoL [14]. However, a common limitation in the literature is the limited preoperative QoL assessments, making it difficult to assess the true extent of postoperative changes. It is important that clinicians and patients are aware of the changes in the QoL post-glossectomy. It helps to ensure realistic expectations are set and guides rehabilitation methods.

To date, no systematic review and meta-analysis exists that comprehensively examines changes in patients’ QoL after undergoing glossectomy. Therefore, this systematic review and meta-analysis aimed to evaluate the post-operative head and neck-related QoL and pre-post operative change in the QoL of tongue cancer patients who underwent glossectomy.

## Methods

This study was conducted following the Preferred Reporting Items for Systematic Reviews and Meta-Analyses (PRISMA) reporting guideline. The study’s protocol was also preregistered in PROSPERO (CRD420251054521).

### Search strategy

The database search was designed and conducted in consultation with a health sciences librarian. We searched PubMed/MEDLINE, Scopus, Web of Science, and Embase. The search strategy was designed to identify studies that assessed the QoL of tongue patients before and/or after glossectomy. The searches included terms for tongue cancer, QoL, and glossectomy (eTable 1, Supplement). Controlled vocabulary terms were included when available. No date limits were applied. Searches were not restricted by publication year. The initial search was conducted on April 1^st,^ 2025, and updated on June 10th, 2025.

### Eligibility and study selection

Inclusion and exclusion criteria were predefined to identify potential studies for this systematic review and meta-analysis. Articles were included in this study if they met the following criteria: (1) reported postoperative QoL outcomes, and preoperative QoL outcomes when available; (2) utilized a validated head and neck-specific QoL assessment tool/scale; (3) case series, cross-sectional studies, or observational studies with either prospective or retrospective designs; (4) were published in peer-reviewed journals; and (5) were published in the English language.

Articles were excluded if they (1) included a patient population composed of multiple head and neck cancer subtypes without explicitly reporting QoL outcomes specific to tongue cancer patients who have undergone glossectomy; (2) were meta-analysis/systematic review, book chapters, Master’s, doctorates, editorials, reviews; (3) were published in languages other than English; and (4) were studies where QoL data could not be sufficiently extracted from text, tables, or figures.

Four investigators (S.T., P.O., O.N., and O.A.) independently screened the titles, abstracts, and full texts in pairs to identify studies that met inclusion criteria. Discrepancies were resolved by consensus among the pair or a third reviewer. Covidence software was used for the screening and study selection process.

### Data extraction

Four investigators (S.T., P.O., O.N., and O.A.) independently extracted data in pairs using the extraction function on the Covidence software. Extracted data included title of the study; first author’s name; country where the study was conducted; study design; total sample size; glossectomy types; reconstruction types (if available); flap reconstruction types (if available); adjuvant radiotherapy; percentage of patients who had radiotherapy; if the study reported pre-post QoL or only post QoL; time interview between surgery and post glossectomy QoL assessment (median follow-up time was used if the duration varies by patient); stage of tongue cancer at surgery (early, advanced, both); types of QoL validated scale or tool used in the study; mean age; percentage of males in the study; and mean (and standard deviation) of QoL scale (composite and subdomains). Disagreements were resolved through consensus between the pair of raters or by a third rater. Data extraction forms were sent to the corresponding authors of the included studies, when necessary, with a request to provide missing data and the option to make corrections, if applicable. Missing standard deviations were calculated from the provided p-values or by using the mean standard deviation of the other studies that will be included.

### Outcome

The primary outcomes assessed in this meta-analysis were postoperative QoL following glossectomy and pre- to postoperative changes in QoL among patients with tongue cancer. Head and neck-related QoL outcomes were measured using validated and standardized QoL instruments/scales. Some of the scales utilized in the included studies comprised the University of Washington Quality of Life (UW-QoL) [15], MD Anderson Dysphagia Inventory (MDADI) [16], Oral Health Impact Profile (OHIP-49) [17], European Organization for Research and Treatment of Cancer Quality of Life Questionnaire-Head and Neck Cancer Module (EORTC QLQ-H&N35) [18], Functional Assessment of Cancer Therapy – Head & Neck (FACT-H&N) [19], Quality of Life in Swallowing Disorders (SWAL-QOL) [20], and the Geriatric Oral Health Assessment Index (GOHAI) [21].

To facilitate comparability across studies, composite QoL scores (standardized to a 0–100 scale when applicable) were derived for each instrument. Specific methods for composite score calculation included: (1) *EORTC QLQ-H&N35*: Sum of scores from all 18 symptom and function domains; (2) *UW-QoL*: Mean of the 12 domain scores, excluding the three global questions; (3) *MDADI*: Mean of the 19 items multiplied by 20; (4) *OHIP-49*: Sum of scores across all 49 items; *(5)* FACT-H&N: Total score derived from the sum of five subscales: Physical, Social/Family, Emotional, Functional Well-Being, and Head and Neck Cancer Subscale; (6) *SWAL-QOL*: Mean score across its 10 subscales. Except for the EORTC QLQ-H&N35, higher scores on the QoL instruments indicated better quality of life, while lower scores reflected poorer outcomes. As the QLQ-H&N35 is scored such that higher values represent greater symptom burden (i.e., worse QoL), its scores were reversed to ensure consistency in interpretation across all instruments.

This standardized approach enabled consistent interpretation and comparison of QoL outcomes across diverse scales. In addition to composite scores, subscale scores (when available) were extracted and assessed for the three most reported QoL challenges among tongue cancer patients: swallowing, speech, and taste.

### Risk of bias

A methodological quality assessment of the included studies was performed using the National Institute of Health (NIH) Quality Assessment for before-after (pre-post) studies with no control group, for cross-sectional studies, and case series. For example, methodological quality in cross-sectional studies was rated as 0 for poor (0–4 out of 14 questions), 1 for fair (5–10 out of 14 questions), or 2 for good (11–14 out of 14 questions). The risk of bias assessment was done by two pairs of reviewers. Disagreements were resolved through consensus between the pair of raters or by a third rater.

### Data analysis

In the meta-analysis of postoperative QoL following glossectomy, forest plots were constructed to visualize the pooled mean estimates of composite QoL, scale-specific QoL, and the three most frequently reported head and neck-related functional impairments: swallowing, speech, and taste. For the pre–post analysis, aggregated means and standard deviations (SDs) of pre- and post-glossectomy QoL scores were utilized. Standardized mean differences (SMDs) were calculated using Hedges’ g (as a small-sample correction to Cohen’s d) to compare pre- and post-glossectomy composite QoL across studies. In addition, weighted mean differences (WMDs) were computed for individual scale QoL, such as the UW-QoL scale.

Each pooled estimate was reported with a corresponding 95% CI. All statistical analyses were performed using a random-effects model employing the restricted maximum likelihood (REML) method to account for between-study variability. Heterogeneity among studies was quantified using the I² statistic, which describes the percentage of total variation across studies attributable to heterogeneity rather than chance [22]. Publication bias was examined and visualized using a funnel plot, and the asymmetry of the plot was tested by Egger’s regression test using the R package “dmetar” [23].

Subgroup meta-analyses were conducted to explore potential sources of heterogeneity based on key clinical and methodological variables. Subgroups included: (1) type of glossectomy: partial/hemi vs. subtotal/near-total/total; (2) reconstruction method: primary closure vs. flap reconstruction; (3) flap reconstruction type: radial forearm free flap (RFF), pectoralis major myocutaneous flap (PMMF), anterolateral thigh free flap (ALTF), and submental island flap (SIF); (4) risk of bias: categorized as fair vs. good quality studies, and (5) stage of the tongue cancer patients (early vs. advanced vs. both). In addition, a univariate meta-regression analysis was performed for continuous moderator variables, including mean patient age, proportion of male participants, follow-up duration, and proportion of patients receiving radiotherapy, to further investigate sources of variability across studies. Sensitivity analysis was performed using the leave-one-out and cumulative meta-analysis.

Statistical analyses were performed using “meta”, “metaviz”, and “dmetar” packages on R Studio version 4.5.1, with *P* < 0.05 from 2-sided tests indicating statistical significance.

## Results

### Study selection

**Fig. 1:**
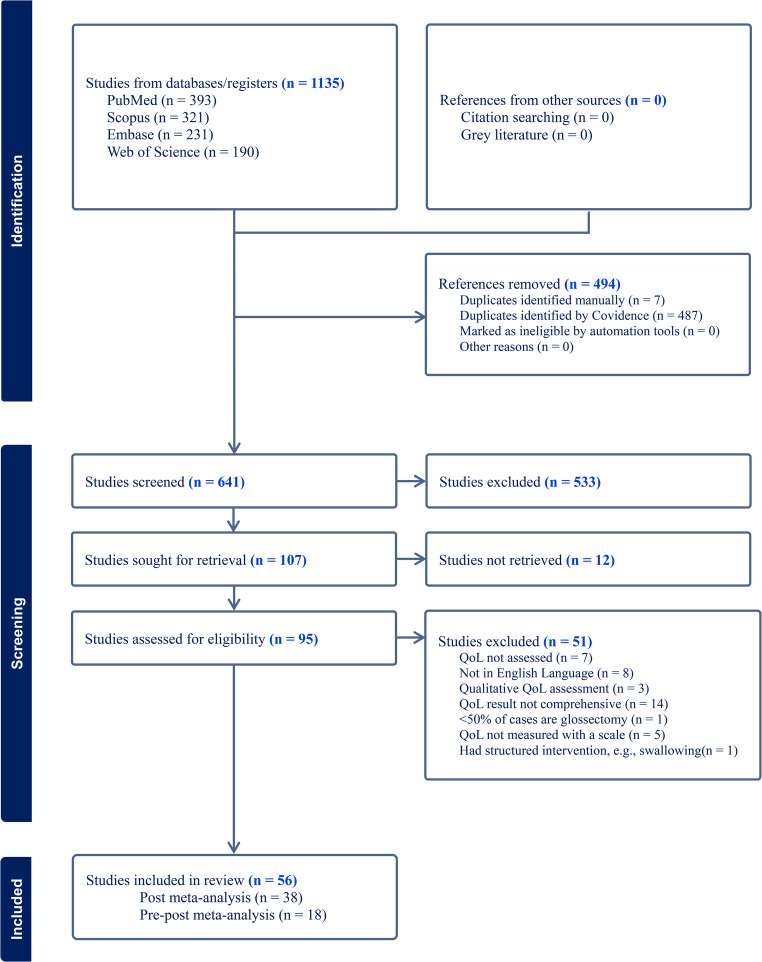
PRISMA flow diagram of literature search and study selection

A total of 1,135 studies were initially identified through database searches. After removing 494 duplicates, 641 unique records remained for screening. During title and abstract screening, 533 studies were excluded due to irrelevance to the study objectives or failure to meet the predefined inclusion criteria. Full-text retrieval was attempted for the remaining 107 articles, of which 12 were excluded as they were conference abstracts without full-text availability. Subsequently, 95 full-text articles were assessed for eligibility, and 51 were excluded for the following reasons: QoL not explicitly assessed (*n* = 7); QoL evaluated using qualitative methods (*n* = 7); incomplete or non-comprehensive QoL reporting (*n* = 14); QoL not assessed using a validated scale (*n* = 5); less than 50% of the patient population had undergone glossectomy (*n* = 1); and full-text not available in English (*n* = 8). Ultimately, 56 studies met the eligibility criteria and were included in the meta-analysis. All included studies reported post-glossectomy QoL outcomes, and 18 of the 56 studies additionally provided pre-glossectomy QoL data ***(***Fig. 1***)***.

### Characteristics of included studies

A total of 2,832 tongue cancer patients who had undergone a form of glossectomy were included across the 56 studies [10, 24–78]. The average age of participants was 55.1 years (± 7.5), and 68.2% were male (1,932 out of 2,832). The included studies were published between 2004 and 2025. The studies originated from diverse global regions. The largest number of studies were from China (*n* = 14), followed by Japan (*n* = 7), India (*n* = 7), Italy (*n* = 6), United Kingdom (*n* = 6), and United States (*n* = 4), with additional contributions from Brazil, Canada, and Germany (*n* = 2 each), and other countries (*n* = 6). With respect to study design, 33 studies were cross-sectional, 21 were prospective or retrospective observational studies, and 3 were case series. Regarding disease stage, 10 studies enrolled patients with early-stage tongue cancer, 8 focused on advanced-stage disease, and the remaining 36 included a combination of early and advanced-stage patients. Most studies (46 of 56) had a good methodological quality, while the remaining studies (10 of 56) had fair methodological quality.

A range of validated instruments/scales was used to assess head and neck-related QoL. The UW-QoL scale was used in 23 studies, followed by the MDADI in 18, and the EORTC QLQ-H&N35 in 13. Additional scales used included the OHIP-49 in 4 studies, FACT-H&N in 5, SWAL-QOL, and GOHAI in one study each. Subdomain QoL scores were reported for swallowing in 45 studies, speech in 32 studies, and taste in 21 studies, representing the most impacted functional domains following glossectomy. More than half of the included studies reported postoperative QoL assessments at 12 months, with a median follow-up time of 12 months. Pre- and post-glossectomy QoL data were available in 18 studies [27, 29, 32, 36, 37, 43, 45, 49, 56, 57, 60–63, 67–69, 73]. In terms of surgical details, 30 studies reported QoL outcomes according to specific glossectomy types, and 31 studies provided stratified outcomes for reconstruction method (primary closure vs. flap reconstruction). Furthermore, 18 studies assessed QoL by specific flap type, including RFF, ALTF, PMMF, and SIF. Table 1 provides further details of the included studies.

**Table 1:** Characteristics of the included studies

Study	Year	Country	Sample size	Glossectomy types	QoL scales	Cancer Stage	Risk of bias assessment
Reported Post QoL Only							
Aimaijiang 2016	2016	Japan	8	Hemi and partial (8)	GOHAI	Early	Good
Amechi 2023	2023	USA	4	Total (4)	UW-QoL, MDADI	Advanced	Good
Boyapati 2013	2013	Ireland	38	Partial (38)	UW-QoL	Early	Good
Canis 2016	2016	Germany	40	Partial (40)	EORTCQLQ-H&N35	Advanced	Good
Chepeha 2023	2023	Canada	101	All types (37 Partial, 33 Hemi, 16 Near-total, 15 Total)	MDADI	Mixed	Good
CostaBandeira 2008	2008	Brazil	29	All types (25 Partial/Hemi, 4 Total)	SWAL-QoL	Mixed	Good
Crombie 2014	2014	Australia	8	All types	EORTCQLQ-H&N35	Mixed	Fair
DeVirgilio 2021	2021	Italy	9	Hemi (5) and Subtotal (4)	EORTCQLQ-H&N35	Mixed	Good
El-Shabrawi 2023	2023	Germany	55	Tongue resection (types no stated)	MDADI, EORTCQLQ-H&N35	Early	Good
Ellabban 2025	2025	England	20	Partial	UW-QoL	Early	Fair
Fang 2013	2013	China	21	All types	UW-QoL	Mixed	Good
Gazzini 2024	2024	Italy	5	Subtotal	MDADI	Advanced	Good
Hartl 2009	2009	France	9	All types (4 Partial/Hemi, 5 Near/Total)	EORTCQLQ-H&N35	Advanced	Good
Kazi 2008	2008	United Kingdom	34	Partial (34)	UW-QoL		Good
Kazi 2008	2008	United Kingdom	31	Partial (28) and Hemi (3)	MDADI	Mixed	Good
Kobayashi 2016	2016	Japan	42	All types	UW-QoL	Advanced	Good
Lazarus 2013	2013	USA	25	Partial (25)	MDADI	Mixed	Fair
Li 2013	2013	China	26	Tongue resection (types no stated)	UW-QoL	Mixed	Fair
Li 2016	2016	China	41	All types	UW-QoL, OHIP-14	Mixed	Good
Liang 2015	2015	China	65	Tongue resection (types no stated)	UW-QoL	Mixed	Good
Mantelakis 2021	2021	United Kingdom	6	Hemi (1) and Partial (5)	EORTCQLQ-H&N35	Mixed	Good
Nemade 2025	2025	India	25	Total (25)	EORTCQLQ-H&N35	Advanced	Good
Ochoa 2021	2021	USA	39	Partial (39)	UW-QoL	Early	Fair
Riva 2022	2022	Italy	22	Partial (22)	EORTCQLQ-H&N35, MDADI	Early	Good
Sakthivel 2017	2017	India	36	Partial (36)	UW-QoL	Early	Fair
Shirakawa 2024	2024	Japan	35	All types (11 Partial, 16 Hemi, 8 Total)	OHIP-14	Mixed	Good
Singh 2025	2025	India	16	Partial and Hemi (16)	UW-QoL	Early	Good
Tonsbeek 2025	2025	Netherlands	77	All types (66 Partial/Hemi, 11 Subtotal/total)	FACT_H&N	Mixed	Good
Wang 2013	2013	China	46	All types	OHIP-14	Mixed	Good
Winter 2004	2004	United Kingdom	12	Tongue resection (types no stated)	UW-QoL	Mixed	Fair
Yuan 2016	2016	China	67	Subtotal (51) and Total (46)	UW-QoL, OHIP-14	Mixed	Good
Zhang 2020	2020	China	190	All types	UW-QoL	Mixed	Good
Zhang 2018	2018	China	90	Hemi (90)	UW-QoL	Mixed	Good
Zhang 2020	2020	China	65	Near Total and Total (65)	UW-QoL, OHIP-14	Mixed	Good
Zhang 2013	2013	China	65	All types	UW-QoL	Mixed	Fair
Massarelli 2023	2023	Italy	52	Tongue resection (types no stated)	FACT_H&N	Mixed	Good
Gabriele 2020	2020	Italy	14	Partial (7) and Hemi (7)	EORTCQLQ-H&N35, MDADI	Mixed	Good
Reported Pre-Post QoL							
Agarwal 2014	2014	India	39	Partial (39)	UW-QoL	Early	Good
Biazevic 2010	2010	Brazil	25	Partial and Total	UW-QoL	Mixed	Good
Brown 2006	2006	England	118	All types	UW-QoL	Mixed	Fair
Cohen 2016	2016	USA	27	All types (19 Partial, 8 Total)	EORTCQLQ-H&N35	Mixed	Good
Dzioba 2017	2017	Canada, Finland, USA	117	Partial (117)	MDADI, EORTCQLQ-H&N35	Mixed	Good
Harada 2024	2024	Japan	40	Hemi (4) and partial (34)	EORTCQLQ-H&N35	Mixed	Good
Ihara 2021	2021	Japan	31	All types (24 Partial/Hemi, 7 Near/Total)	EORTCQLQ-H&N35	Mixed	Good
Koizumi 2013	2013	Japan	26	All types	FACT_H&N	Mixed	Fair
Mercante 2015	2015	Italy	13	Tongue resection (types no stated)	MDADI	Early	Good
Mitra 2024	2024	India	24	Subtotal (17) and Total (7)	MDADI, FACT_H&N	Advanced	Good
Ou 2023	2023	China	138	All types	MDADI	Both	Good
Pyne 2020	2020	Canada	22	Total	MDADI	Advanced	Good
Rios-Gonzalez 2024	2024	Spain	44	All types (25 Partial, 5 Hemi, 6 Total)	UW-QoL	Mixed	Good
Suzuki 2023	2023	Japan	43	Hemi (19) and Total (24)	SF-8	Mixed	Good
Tamer 2020	2020	China	265	Partial (205) and Hemi (60)	FACT_H&N, MDADI	Mixed	Good
Yang 2010	2010	China	231	Tongue resection (types no stated)	UW-QoL	Mixed	Good
Deng 2022	2022	China	67	Hemi (50) and Total (17)	MDADI	Mixed	Good
Ravindra 2022	2022	India	47	Partial and Hemi (47)	MDADI	Mixed	Good
Sundram 2024	2024	India	47	All types (14 Partial, 27 Hemi, 3 Subtotal, 3 Total)	EORTCQLQ-H&N35, MDADI	Mixed	Good

### QoL post-glossectomy

The current meta-analysis estimated a pooled composite QoL score of 71.9% (95% CI: 68.3–75.4; I² = 99.8%) following glossectomy (Table 2), with no evidence of publication bias (eFigure 1, Supplement). Domain-specific pooled mean QoL scores were 66.8% for swallowing (95% CI: 61.1–72.5; I² = 99.0%), 70.7% for speech (95% CI: 65.1–76.0; I² = 99.9%), and 70.6% for taste (95% CI: 64.8–76.4; I² = 98.6%).

**Table 2:** Results of meta-analysis of quality-of-life post-glossectomy (overall, by QoL scales, glossectomy types, reconstruction options, and flap types)

	No. of studies	Mean	95% CI
**All studies** Overall composite QoL Swallowing Speech Taste	56 45 32 21	71.9 66.8 70.7 70.6	68.3–75.4 61.1–72.5 65.1–76.0 64.8–76.4
Different scales EORTCQLQ-H&N35 UW-QoL MDADI FACT H&*N*	13 23 18 5	84.8 72.9 63.1 75.6	77.8–91.9 68.5–77.2 52.7–73.4 54.2–96.9
**Glossectomy types** Partial/Hemi glossectomy Composite QoL Swallowing Speech Taste Subtotal/Near total/Total glossectomy Composite QoL Swallowing Speech Taste	23 17 15 8 15 11 6 2	76.3 73.6 75.8 79.2 66.3 66.4 56.1 60.6	70.8–81.8 63.4–83.7 65.7–85.9 71.6–86.7 59.8–72.7 57.6–75.2 37.8–74.3 51.- − 70.1
**Reconstruction options** Primary closure Composite QoL Swallowing Speech Taste Flap reconstruction (all) Composite QoL Swallowing Speech Taste Flap reconstruction (assessment done after 12 months) Composite QoL Swallowing Speech Taste	8 6 5 1 30 23 15 8 8 8 5 2	81.1 74.6 80.0 94.4 68.1 64.1 68.8 68.2 68.7 57.0 67.7 66.2	74.6–87.6 64.5–84.7 71.1–88.9 88.3–100.0 63.0–73.3 56.4–71.7 62.3–75.2 58.9–77.5 57.6–79.8 40.7–73.5 58.5–76.9 50.7–81.6
**Partial/Hemi glossectomy only** Primary closure Flap reconstruction (all) Flap reconstruction (assessed > 12 mo)	2 14 4	80.8 69.2 68.4	57.1–99.9 61.6–76.7 48.6–88.3
**Flap types** RFF PMMF ALTF SIF	7 3 9 2	73.6 67.9 64.4 64.8	64.9–82.2 57.4–78.3 53.8–75.0 60.1–69.5

**Fig. 2:**
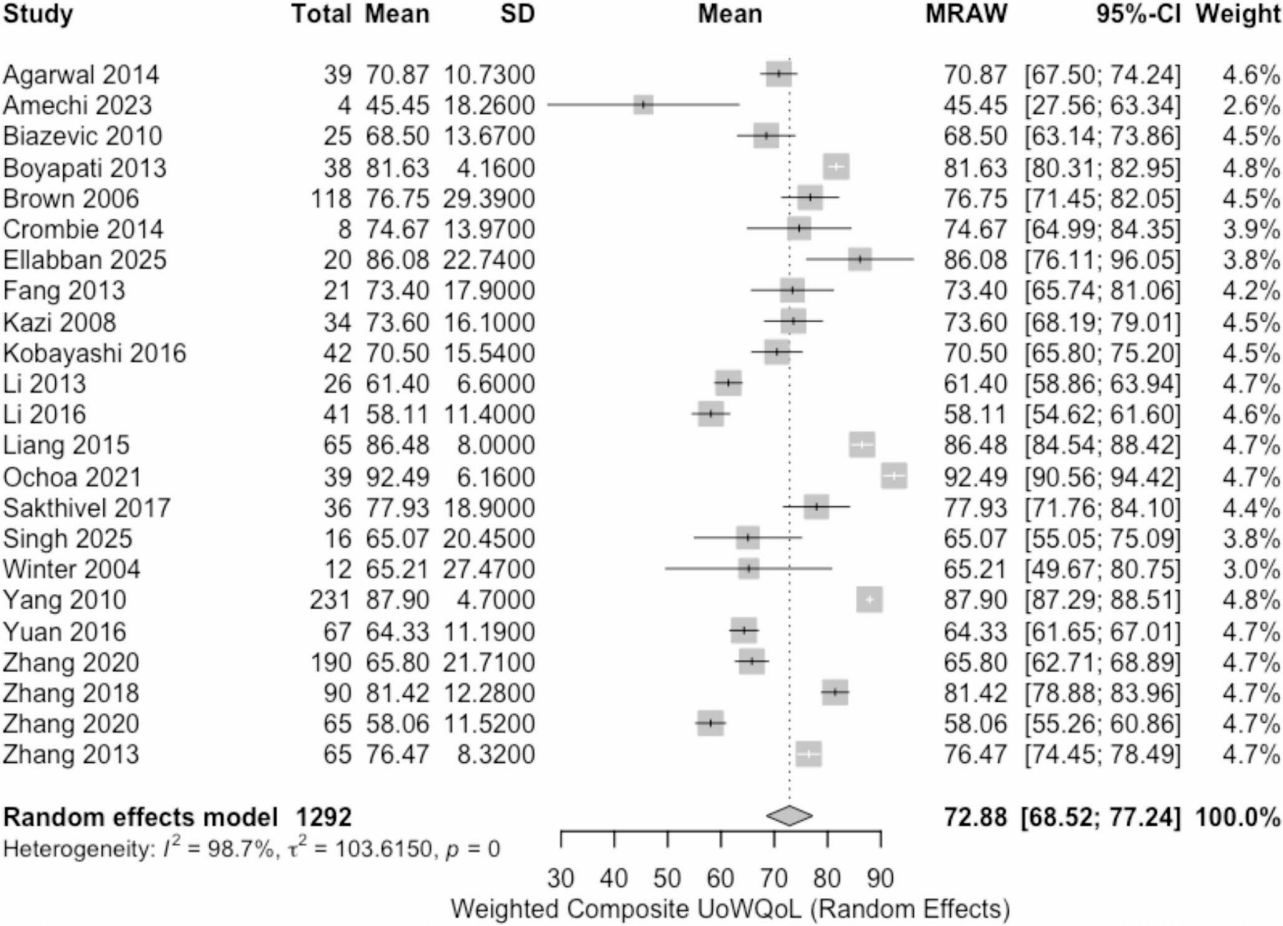
Forest plot of quality-of-life post-glossectomy weighted (UWQoL scale)

When analyzed by individual QoL instruments, the EORTC QLQ-H&N35 demonstrated the highest pooled post-glossectomy QoL score (84.8%; 95% CI: 77.8–91.9; I² = 95.8%), followed by the FACT-H&N (75.6%; 95% CI: 54.2–96.9; I² = 99.6%), UW-QoL (72.9%; 95% CI: 68.5–77.2; I² = 98.7%, Fig. 2), and MDADI (63.1%; 95% CI: 52.7–73.4; I² = 99.9%) (Table 2).

Subgroup analyses revealed that patients who underwent partial or hemi-glossectomy had significantly higher post-glossectomy composite and domain-specific QoL scores (composite mean = 76.3%; 95% CI: 70.8–81.8; I² = 99.6%) compared to those who underwent subtotal, near-total, or total glossectomy (composite mean = 66.3%; 95% CI: 59.8–72.7; I² = 90.3%) (Table 2; eFigures 2 and 3, Supplement). Similarly, patients who received primary closure reported better QoL outcomes across composite, swallowing, speech, and taste domains than those who underwent flap reconstruction (Table 2; eFigures 4 and 5, Supplement). Specifically, the composite QoL for patients who underwent free flap reconstruction and were assessed after 12 months was 76.3% (95% CI: 57.6–79.8; I² = 99.4%) ***(***Table 2***)***. Among those who underwent partial or hemi-glossectomy, patients who received primary closure had better QoL (composite mean = 80.8%; 95% CI: 57.1–72.7; I² = 96.3%) compared to those who received free flap closure (composite mean = 69.2%; 95% CI: 61.6–76.7; I² = 98.7%) ***(***Table 2***)***. Among flap types, QoL was highest for patients reconstructed with RFF, followed by PMMF, ALTF, and SIF ***(***Table 2***)***.

Sensitivity analyses, including cumulative and leave-one-out approaches, demonstrated that the exclusion of any single study did not significantly affect the overall pooled QoL estimates. In univariate meta-regression, a statistically significant association was observed between composite QoL and the percentage of male participants in the included studies (estimate = −0.38; 95% CI: −0.59 to −0.17; *p* < 0.001, eFigure 6, Supplement). However, no significant associations were identified for year of publication, mean age, follow-up duration, or the proportion of patients who received radiotherapy.

### Change in QoL Pre-Post glossectomy

**Fig. 3:**
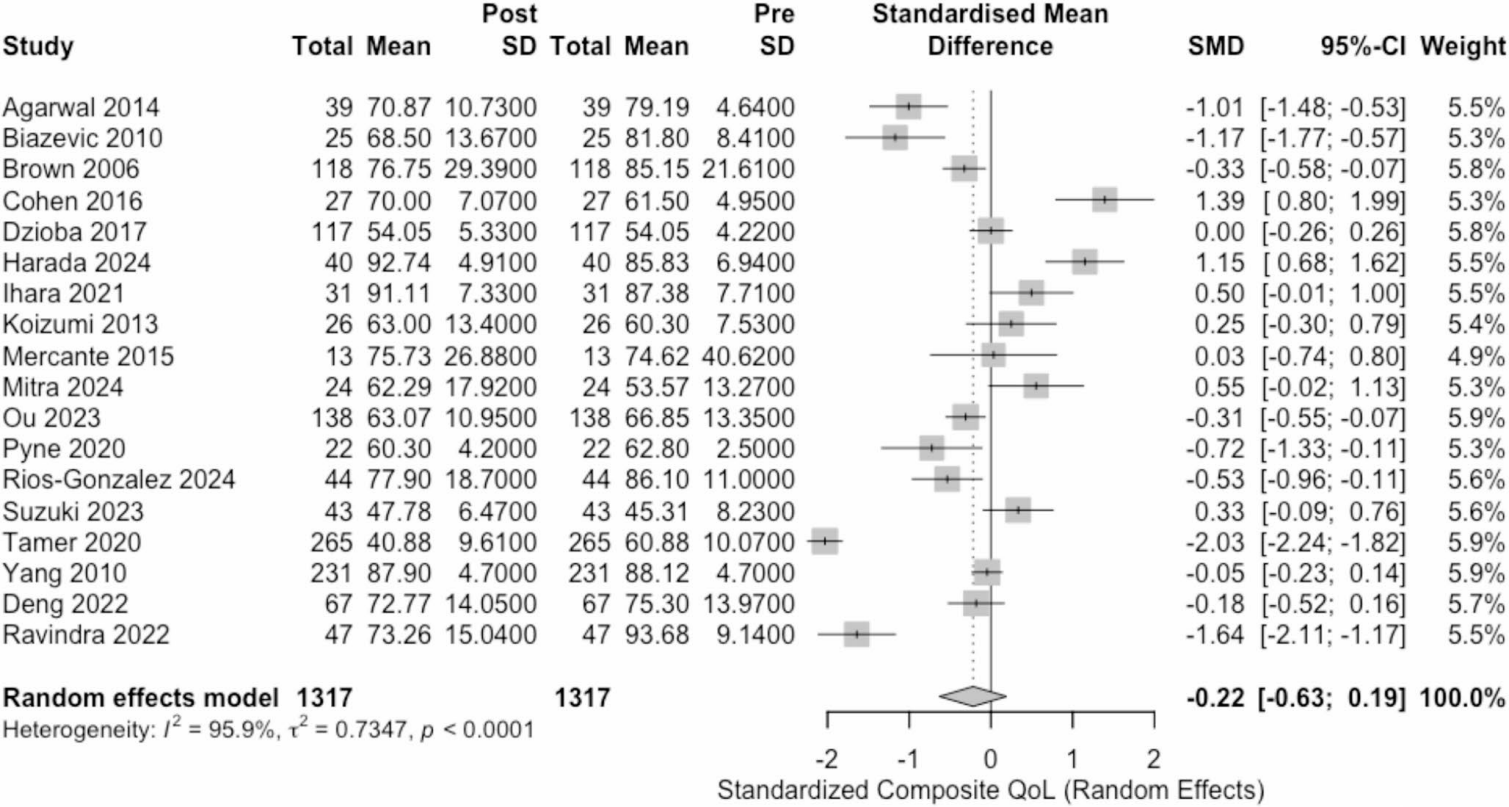
Forest plot of the change in composite quality-of-life before and after glossectomy

The meta-analysis of 18 studies reporting both pre- and post-glossectomy QoL found an overall stabilization in QoL, with no significant change observed in composite or most subdomain scores. Specifically, the SMD for composite QoL was − 0.22 (95% CI: −0.63 to 0.19; I² = 95.9%, Fig. 3), indicating no statistically significant difference between pre- and post-glossectomy QoL scores. Similarly, subdomain analyses revealed non-significant changes in swallowing (SMD = 0.30; 95% CI: −1.18 to 1.77; I² = 98.0%) and speech (SMD = −0.62; 95% CI: −2.53 to 1.29; I² = 98.2%). In contrast, taste function showed a significant decline in QoL following glossectomy (SMD = −1.72; 95% CI: −3.07 to −0.38; I² = 95.5%) ***(***Table 3***)***.

**Table 3:** Results of meta-analysis of the change in quality-of-life before and after glossectomy (overall, by QoL scales, glossectomy types, reconstruction options and flap types)

	No. of studies	SMD	95% CI
All studies Overall composite QoL Swallowing Speech Taste	18 14 7 3	−0.22 0.30 −0.62 −1.72	−0.63–0.19 −1.18–1.77 −2.53–1.29 −3.07 – −0.38
Different scales EORTCQLQ-H&N35 UW-QoL MDADI FACT H&*N*	3 4 9 3	−3.20 −7.01 −4.10 −1.76	−7.72–1.33 −12.68 – −1.34 −12.23–4.03 −21.21–17.70
Glossectomy types Partial/Hemi glossectomy Subtotal/Near total/Total glossectomy	10 7	−0.06 0.03	−0.82–0.69 −0.42–0.48
**Reconstruction options** Primary closure Flap reconstruction	2 7	−0.90 −0.72	−2.66–0.86 −1.78–0.33

Analysis by QoL scale revealed similar stabilization in pre-post QoL across all instruments, except for the UW-QoL scale ***(***Table 3***)***. Patients assessed with the UW-QoL experienced a statistically significant mean decrease of 7.01 points post-glossectomy compared to baseline (WMD = −7.01; 95% CI: −12.68 to −1.34; I² = 92.1%; eFigure 7, Supplement). There was no evidence of publication bias, as indicated by Egger’s test (test statistic = 1.14; *p* = 0.127, eFigure 8, Supplement).

Subgroup analyses showed consistent patterns of QoL stabilization among patients undergoing partial/hemi-glossectomy (eFigure 9, Supplement), subtotal/near-total/total glossectomy (eFigure 10, Supplement), and those who underwent flap reconstruction (eFigure 11, Supplement). Sensitivity analyses, including cumulative and leave-one-out analyses, showed no single study disproportionately influenced the results. Finally, univariate meta-regression analyses found no statistical significance when controlling for year of publication, mean age, percentage of male participants, follow-up duration, or proportion of patients receiving radiotherapy.

## Discussion

The QoL of patients with tongue cancer is affected by the cancer process itself and its treatments, especially surgical procedures like glossectomy [79]. Assessing QoL outcomes related to tongue resection and glossectomy is crucial for shaping patient expectations, directing therapeutic decisions, and enhancing postoperative care and rehabilitation approaches. To the best of our knowledge, this is the first meta-analysis examining the pooled change in QoL of tongue cancer patients pre-post glossectomy stratified by different head and neck-related QoL scales, glossectomy types, reconstruction, and flap types.

The overall composite QoL result suggested that patients with tongue cancer may potentially report a satisfactory QoL after undergoing glossectomy. A similar pattern was found in tongue cancer patients’ three most significant dimensions of QoL: swallowing, speech, and taste. These essential functions depend significantly on the anatomical and neuromuscular integrity of the tongue, which is essential for mastication, articulation, and gustatory perception [80]. Tongue cancer and associated surgical intervention markedly diminish oral function, adversely affecting nutritional intake, vocal communication, and sensory perception, each of which directly influences patients’ psychological health and everyday activities [80, 81].

This meta-analysis, albeit with high heterogeneity, also suggested a vast difference in post-glossectomy QoL between patients who underwent partial or hemi-glossectomy and those who underwent subtotal, near-total, or total glossectomy. Tongue cancer patients who undergo more extensive tongue resections typically have a marked reduction in QoL [45]. This might be because of the increased degree of functional tissue loss, including motor and sensory innervation, and diminished tongue mobility necessary for bolus manipulation, articulation, and gustatory perception [45, 82]. Significant resections frequently disturb the anatomical symmetry of the oral cavity and necessitate more intricate reconstructive interventions, thereby potentially impacting rehabilitation results [83]. In addition, individuals who had partial or hemi-glossectomy and underwent primary closure showed higher overall and domain-specific QoL than those who underwent flap-based reconstruction. Primary closure is generally achievable in minor tongue resections, retaining a greater portion of the original tongue structure, conserving neuromuscular function, and promoting expedited healing [84]. However, flap reconstruction, although essential for extensive defects, frequently incorporates non-native tissue that does not possess the functional characteristics of the tongue (such as muscle tone, sensation, and coordination), which may lead to stiffness, bulkiness, and diminished adaptability in the dynamic oral environment [84, 85].

Among different flap reconstructions explored, the RFF and PMMF showed potential relatively higher QoL than ALTF and SIF. RFF is usually preferred for its thin, flexible, and sensitive tissue, which closely resembles the mobility and shape of the tongue, facilitating improved speech and swallowing results [86]. On the other hand, ALTF or SIF flaps, although beneficial, may result in increased tissue bulk or variability in functional outcomes, especially if sensory reinnervation or mobility is restricted [86].

Overall, there was no statistically significant change in composite QoL levels after glossectomy, according to the pre-post meta-analysis. The high heterogeneity of this meta-analysis suggests caution interpretation of this result. Patients frequently manage to adjust to their postoperative condition over time, despite the functional and anatomical alterations associated with tongue resection. A few factors could cause this stability. First, patients may be able to make up for lost oral function using interdisciplinary and multidisciplinary care that includes rehabilitation, speech therapy, and nutritional support [87]. Second, a sense of well-being that offsets some of the functional deficits can result from the psychological adjustment that happens after treatment, particularly when the cancer is properly managed [88]. Despite the aforementioned reasons, an important consideration in interpreting these findings is the potential role of psychological adaptation and response shift [89]. Even in the presence of persistent functional impairments, such as difficulties with speech, swallowing, or taste, patients may recalibrate their internal standards, reprioritize domains of well-being, or reconceptualize what constitutes an acceptable quality of life. This process can create the appearance of ‘stable’ QoL scores despite ongoing functional decline. Response shift has been described in other cancer populations and likely reflects patients’ ability to derive meaning from survivorship, adjust expectations, and place greater emphasis on psychosocial or emotional domains once the acute threat of cancer is addressed [90]. Recognizing this phenomenon is critical, as it underscores that stable composite QoL scores may not fully capture the lived reality of patients who continue to experience substantial functional deficits.

### Limitations and strengths

Our meta-analysis has several limitations. (1) The high heterogeneity identified in our study warrants a cautious interpretation of the findings. This variability could be a result of the extensive inclusion and exclusion criteria employed in each study, differential study designs, and geographical distribution of the studies. This heterogeneity poses a potential risk of introducing publication bias and may affect the generalizability of the findings. (2) Calculating and combining composite QoL scores did not demonstrate the true picture of the QoL among these patients. Nonetheless, this was necessary for this meta-analysis as the included studies used different QoL scales, with each scale having an average of more than 10 subdomains. However, conceptual differences between these QoL instruments may dilute domain-specific insights, and the resulting composite scores should therefore be interpreted with caution. (3) The use of validated and multidimensional QoL instruments captures a broader spectrum of life experiences (e.g., emotional, social, and physical well-being), which may dilute the apparent impact of functional loss in one domain. (4) The interval between glossectomy and subsequent QoL assessments varied from 1 to 96 months (median: 12 months) in the included studies, and there is typically a change in the patient’s QoL over time following surgery. (5) The majority of the studies were cross-sectional in nature, thereby warranting cautious interpretation of the findings. Furthermore, with only 18 of the 56 included studies reporting pre–post QoL data, the evidence base is insufficient to support strong causal inferences regarding temporal changes. (6) More than 50% of the included studies were from Asia (China, Japan, and India), thereby limiting the global generalizability of the findings due to factors such as cultural and healthcare system differences. (7) Non-English studies were excluded, and gray literature was not searched. (8) Given the limited studies explicitly reporting QoL outcomes in patients who have undergone secondary closure following glossectomy, we were unable to determine whether a difference in QoL exists between primary and secondary closure techniques.

Despite these limitations, this was the first meta-analysis examining the pooled change in QoL of tongue cancer patients pre-post glossectomy stratified by different head and neck-related QoL scales, glossectomy types, reconstruction, and flap types.

## Conclusion

In conclusion, this meta-analysis suggests that patients with tongue cancer may maintain aspects of QoL following glossectomy, particularly in functional domains such as swallowing, speech, and taste. However, given the very high heterogeneity across studies, these findings should be interpreted with caution. QoL outcomes appear to vary considerably depending on the extent of glossectomy and the type of reconstruction. These findings highlight the significance of personalized surgical planning and rehabilitation strategies to maintain function and enhance patient-centered outcomes in the management of tongue cancer.

## Supplementary Information

Below is the link to the electronic supplementary material.

Supplementary Material 1 (DOCX 1.13 MB)

## Data Availability

All the data were extracted from secondary sources and are cited in the manuscript.
