# Supplementary material for: Head and neck related quality of life following glossectomy among tongue cancer patients: a systematic review and meta-analysis
Source: Oral Maxillofac Surg. 2025 Sep 30;29(1):164. doi: 10.1007/s10006-025-01471-y (PMC12479587; doi:10.1007/s10006-025-01471-y)

**SUPPLEMENTARY TABLES AND FIGURES**

**eTable 1: Keywords for study search to identify published articles on quality of life of tongue cancer patients who have undergone glossectomy**

| **Database** | **Search Keywords** |
| --- | --- |
| PubMed/Medline | ("Glossectomy"[Mesh] OR "Tongue Neoplasms"[Mesh] OR “Tongue resection” OR “Partial glossectomy” OR “Subtotal glossectomy” OR “Total glossectomy” OR “Hemiglossectomy” OR “tongue cancer” OR glossectom*) AND ("Quality of Life"[Mesh] OR “quality of life” OR “health-related quality of life” OR “HRQoL”). |
| Scopus | ( TITLE-ABS-KEY ( glossectomy ) AND TITLE-ABS-KEY ( quality AND of AND life ) ) |
| Embase | 'glossectomy'/exp AND 'quality of life'/exp |
| Web of Science | (ALL=(glossectomy)) AND ALL=(quality of life) |

**eFigure 1. Meta-analysis of quality-of-life post-glossectomy: Funnel Plot**


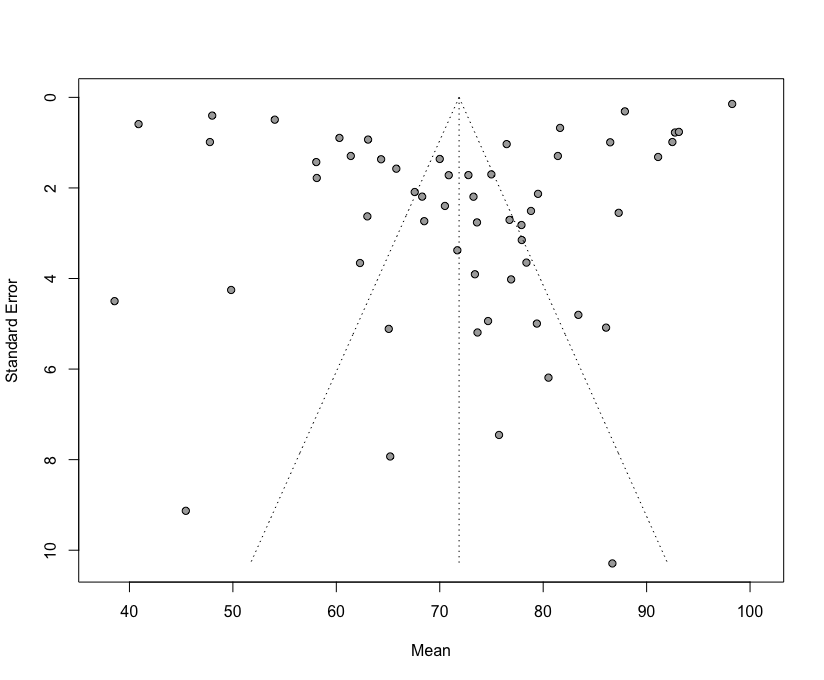


**eFigure 2. Forest plot of quality-of-life post-glossectomy weighted (Partial/Hemi glossectomy patients)**


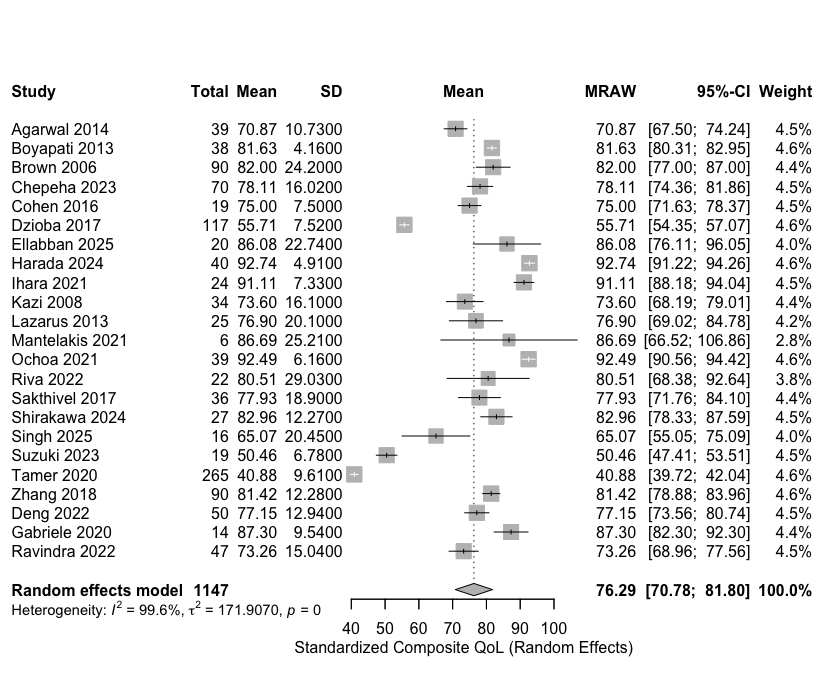


**eFigure 3. Forest plot of quality-of-life post-glossectomy weighted (Subtotal/Near total/ Total glossectomy patients)**


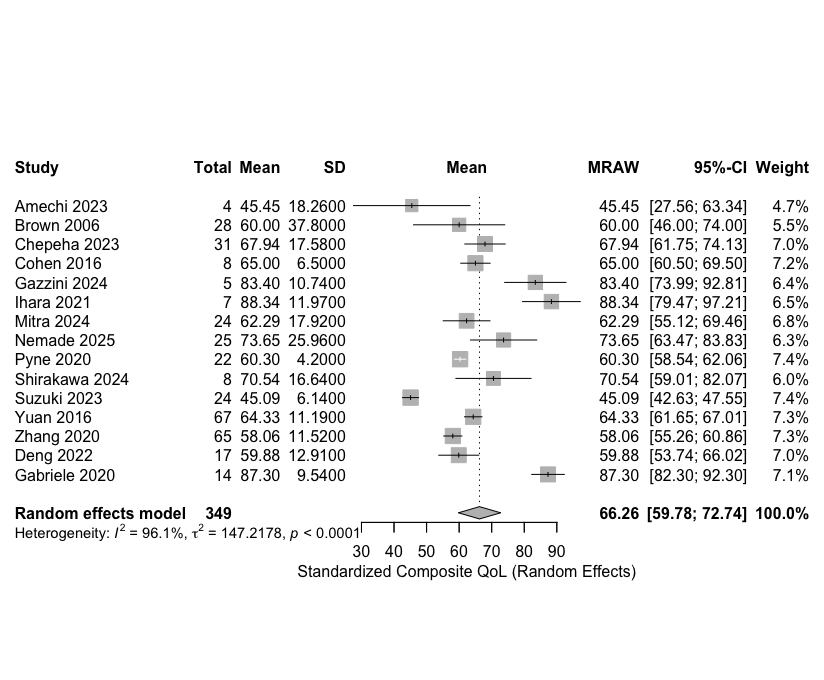


**eFigure 4. Forest plot of quality-of-life post-glossectomy weighted (Primary Closure)**


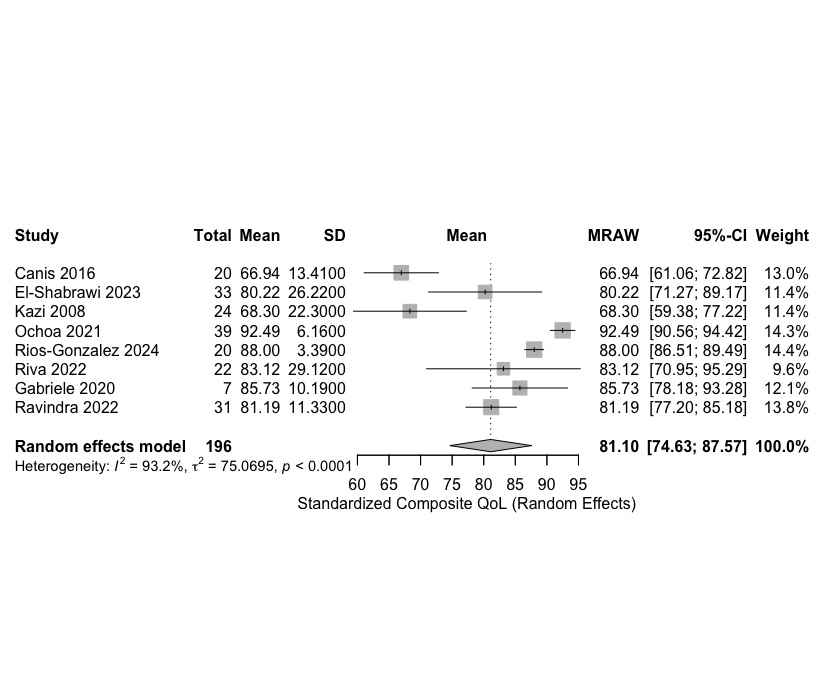


**eFigure 5. Forest plot of quality-of-life post-glossectomy weighted (Flap reconstruction)**


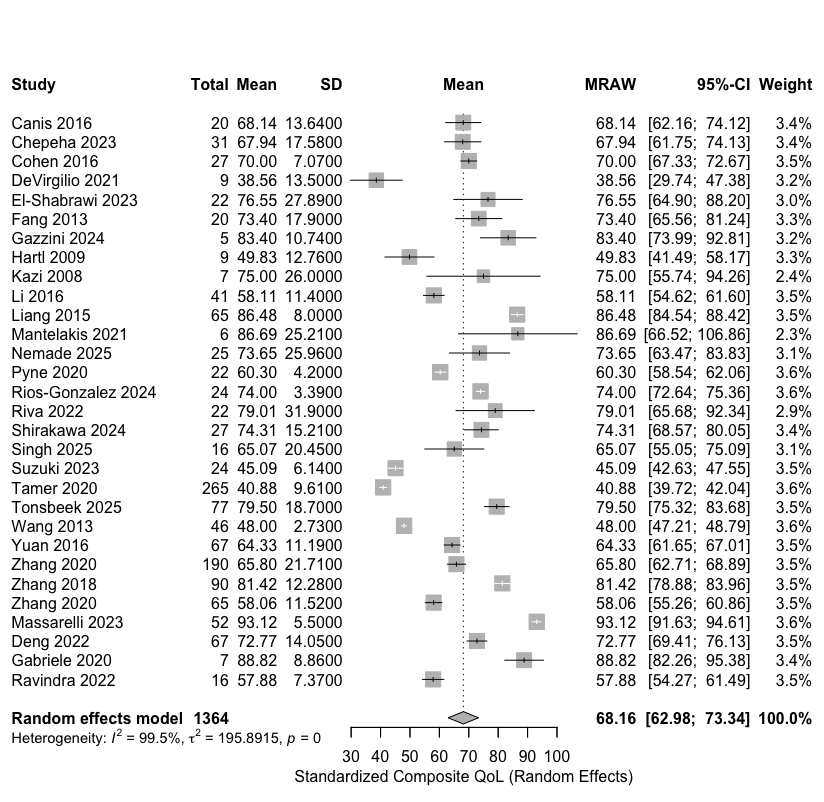


**eFigure 6. Bubble plot for the meta-regression of the quality-of-life post-glossectomy with percentage of male in the studies**


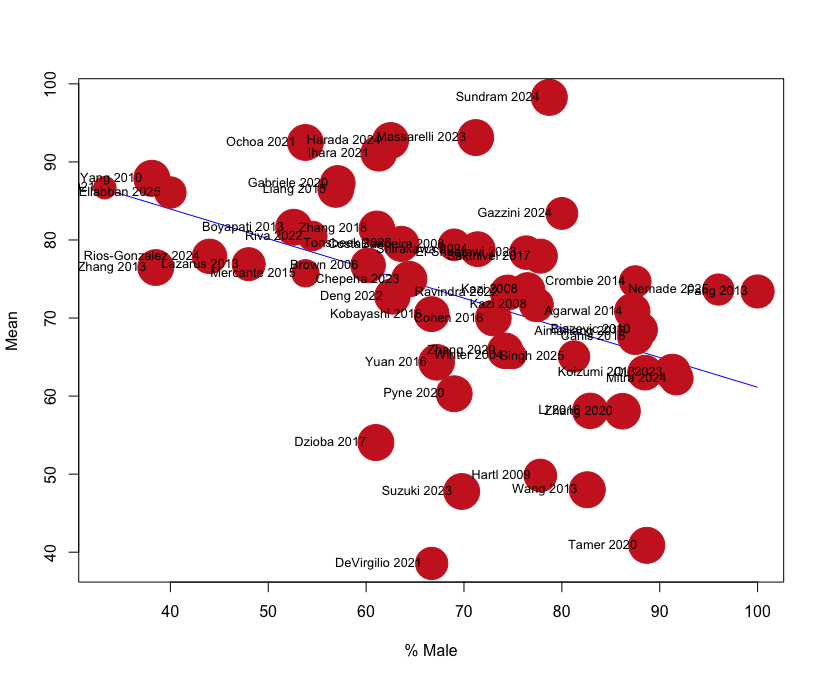


**eFigure 7. Forest plot of the change in quality-of-life before and after glossectomy (UWQoL scale)**


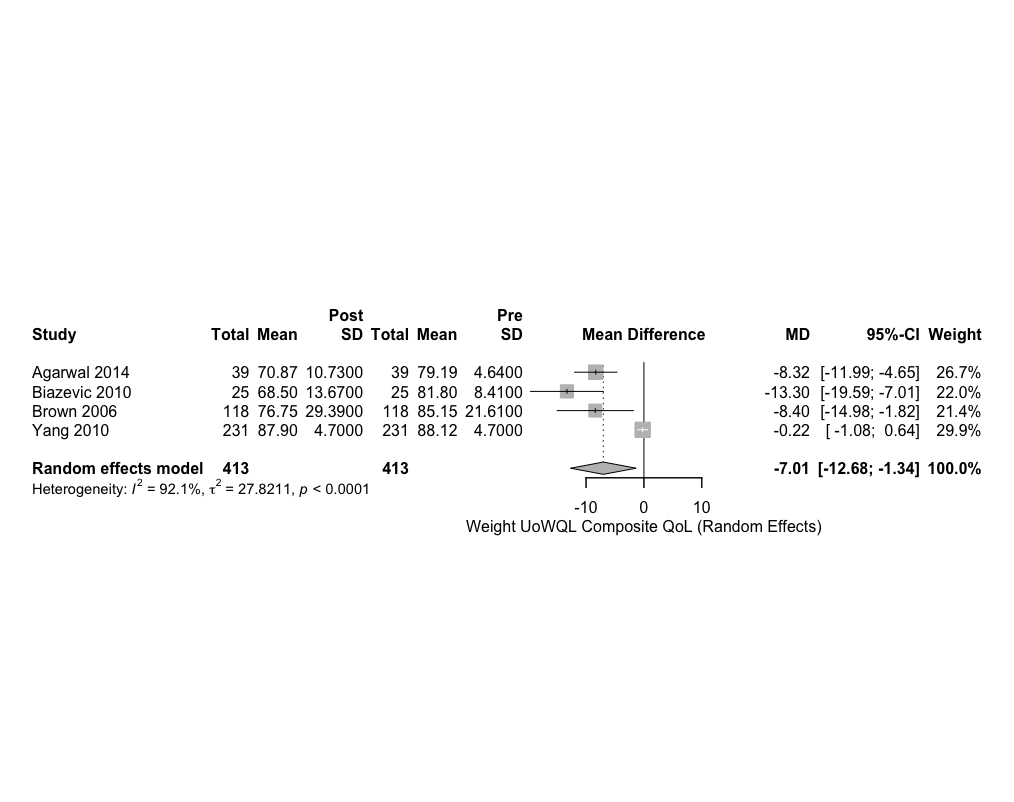


**eFigure 8. Meta-analysis of change in quality-of-life before and after glossectomy: Funnel Plot**


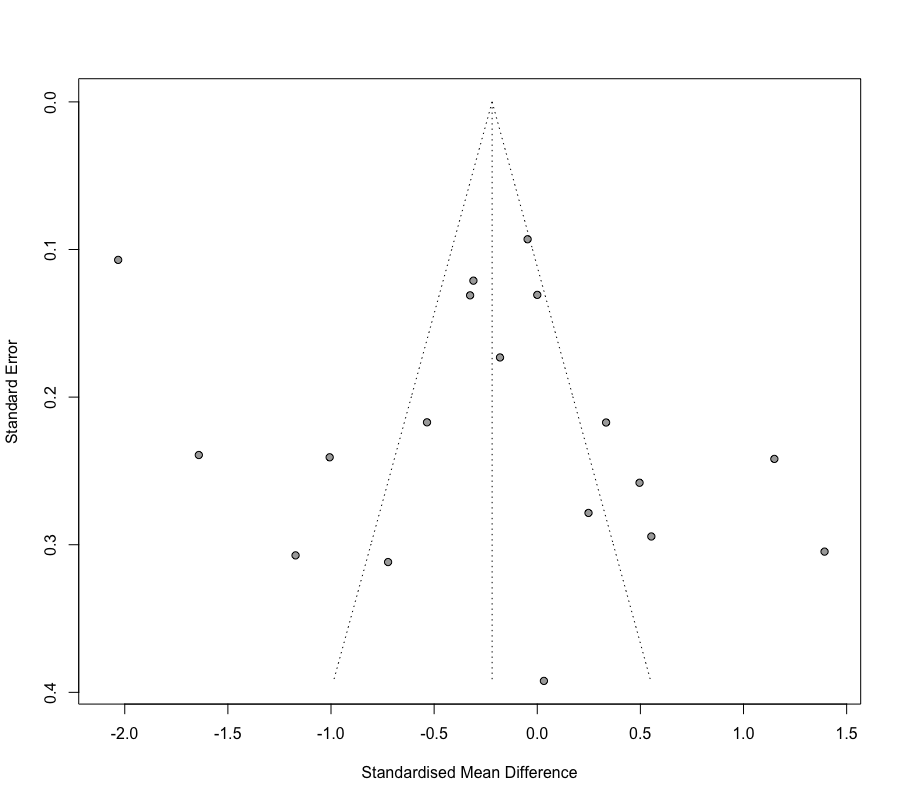


**eFigure 9. Forest plot of the change in composite quality-of-life before and after glossectomy (Partial/Hemi Glossectomy)**


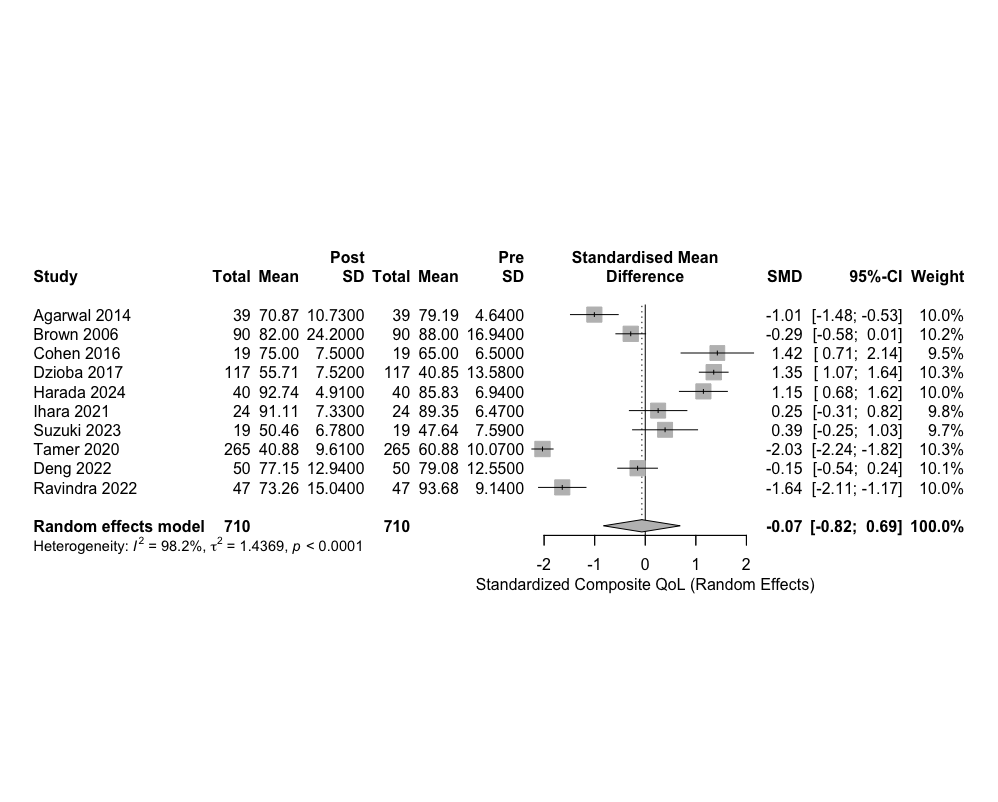


**eFigure 10. Forest plot of the change in composite quality-of-life before and after glossectomy (Subtotal/Near Total/Total Glossectomy)**


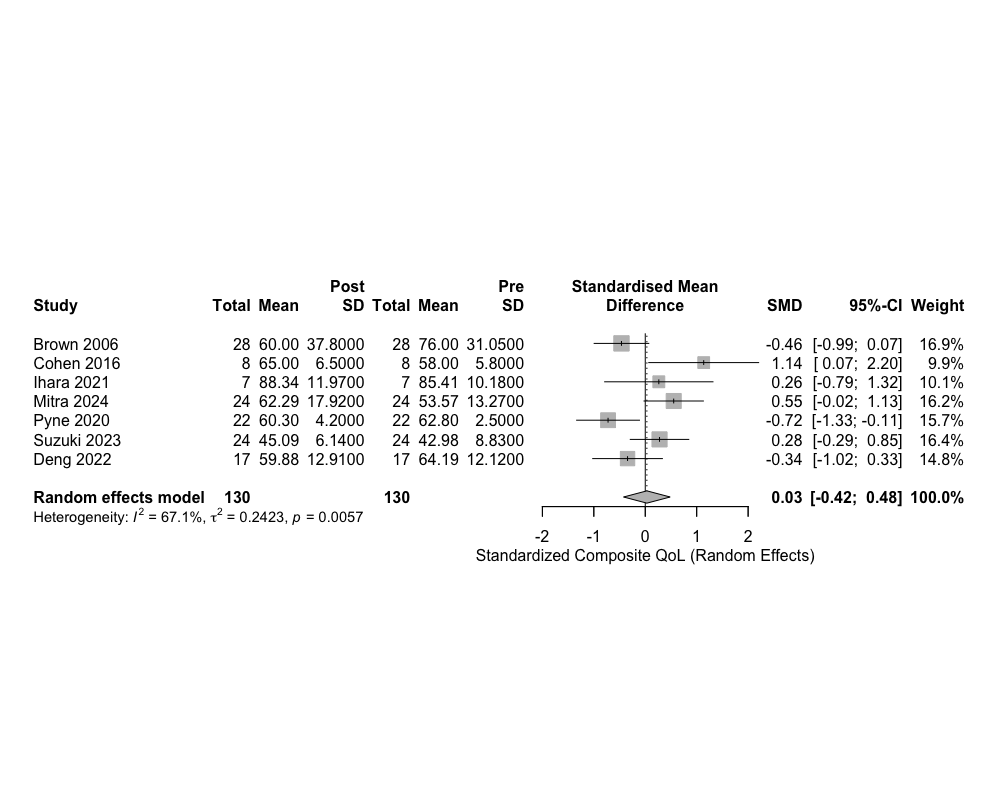


**eFigure 11. Forest plot of the change in composite quality-of-life before and after glossectomy (Flap Reconstruction)**


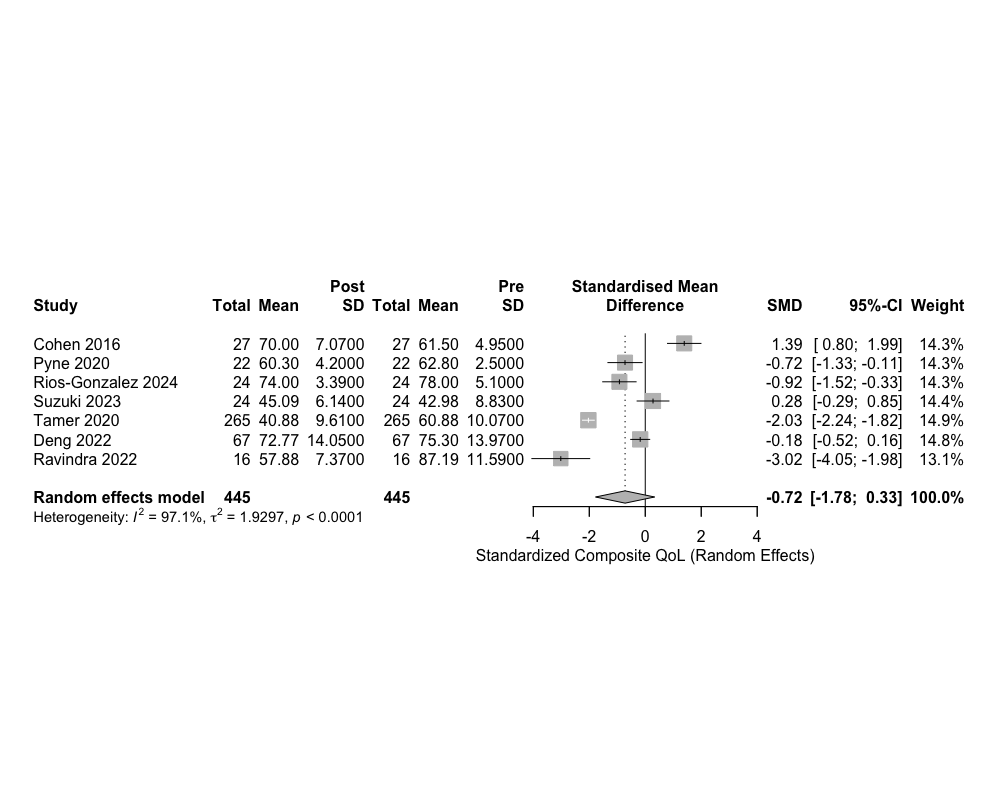

Supplement: Supplementary file 1 — Supplementary Material 1 (DOCX 1.13 MB) [file 10006_2025_1471_MOESM1_ESM.docx]
